# Clinical Efficacy and Safety of Zoledronic Acid Combined with PVP/PKP in the Treatment of Osteoporotic Vertebral Compression Fracture: A Systematic Review and Meta-Analysis of Randomized Controlled Trials

**DOI:** 10.1155/2021/6650358

**Published:** 2021-04-08

**Authors:** Yan Sun, Haoning Ma, Feng Yang, Xiangsheng Tang, Ping Yi, Mingsheng Tan

**Affiliations:** ^1^Beijing University of Chinese Medicine, Beijing 100029, China; ^2^Department of Orthopaedics, China-Japan Friendship Hospital, Beijing 100029, China

## Abstract

**Objective:**

We conducted this meta-analysis to provide better evidence of the efficacy and safety of zoledronic acid (ZA) combined with percutaneous vertebroplasty/kyphoplasty (PVP/PKP) on osteoporotic vertebral compression fracture (OVCF) and proposed a protocol for its application in clinical practice.

**Methods:**

All randomized controlled trials (RCTs) of ZA combined with PVP or PKP compared to individual PVP/PKP for the management of patients with OVCFs were included in this study. Electronic database searches were conducted from database inception to November 2020, including the Cochrane Library, PubMed, Web of Science, and Embase. The pooled data were analyzed using RevMan 5.3 software.

**Results:**

Seven RCTs with 929 subjects were finally included. All included studies reported visual analog scores (VAS), and no statistically significant differences were identified at follow-ups of 3 d and 1 w (*P* > 0.05). In contrast, significant differences were observed at the 1 mo, 3 mo, 6 mo, and 12 mo follow-ups (*P* < 0.05). Two trials reported the Cobb angle and vertebral body height (VBH), including 182 subjects without significant differences at the 12 mo follow-up (*P* > 0.05). In addition, significant differences in the bone mineral density (BMD), *β*-isomerized C-terminal telopeptide of type I collagen (*β*-CTX), N-terminal propeptide of type I collagen (PINP), and N-terminal molecular fragment (N-MID) levels were observed between the two groups (*P* < 0.05). All trials reported side effects. Significant differences in recurrent fractures, fever, flu-like symptoms, and arthralgia or myalgia were identified (*P* < 0.05); however, no significant difference in postoperative leakage was detected (*P* > 0.05).

**Conclusion:**

Compared to PVP/PKP alone, an additional ZA injection had advantages of long-term analgesic effects with improved bone metabolism indexes. Moreover, combination therapy significantly prevented complications and drug reactions were well tolerated. Overall, this systematic review revealed that ZA combined with PVP/PKP was an effective, safe, and comprehensive therapy for patients with OVCFs.

## 1. Introduction

Osteoporosis is a metabolic bone disease with a low bone density that is characterized by systemic skeletal pain and a susceptibility to fracture [1]. Osteoporotic vertebral compression fracture (OVCF), one of the most common complications of osteoporosis, has become an urgent public health issue in aging societies [2]. According to a report, OVCFs affect millions of people worldwide, and the morbidity in elderly individuals could be as high as 11-50% [3, 4]. Patients may experience obvious low back pain with limited mobility, severe kyphosis with a long course, and frequent relapse. Thus, OVCFs affect the physical and mental health and reduces quality of life and even life expectancy.

Initially, conservative treatments were developed for OVCFs, including analgesics, bed rest, physical therapy, and antiresorptive medications. However, the aforementioned management does not address kyphotic deformities, which are the key factor contributing to the spinal biomechanical balance; thus, adjacent vertebral fractures subsequently occur [5]. Moreover, long-term bed rest often has a negative impact on quality of life and societal burdens. In particular, for elderly individuals with a poor physical condition, side effects of oral drugs and analgesics must be carefully considered, along with complications from immobilization and a completely bedridden status [6]. Therefore, the indications for conservative treatment are rather narrow and do not meet the current treatment requirements for OVCFs.

Under these circumstances, PVP and PKP were introduced [7, 8]. To date, PVP has been widely accepted as the gold standard for the treatment of OVCFs due to its advantages in relieving pain and rapidly restoring the height of the corresponding vertebral body. In addition, PKP was reported to be superior for patients with large kyphotic deformities, vertebral fissures, or a substantial height loss in the fractured vertebrae [1]. Surgical procedures have been broadly utilized, although with potentially underestimated problems. Among the main problems is secondary adjacent vertebral fractures. Zhang et al. stated that 12.9% of patients experienced new fractures within 1 year after PVP [9], and Shi et al. reported a 14.7% secondary fracture rate in patients who received PKP [10]. Indeed, the natural progression of osteoporosis and the additional mechanical pressure of injected bone cement contributing to refracture cannot be ignored. Meanwhile, postoperative residual pain obviously affects the quality of life, as PKP/PVP fails to relieve the pain caused by osteoporosis; conversely, the load of the injected bone cement in vertebral bodies may aggravate the discomfort. Apparently, the progression of osteoporosis is the main cause of OVCFs, and surgery alone is an incomplete treatment for OVCFs; accordingly, a systemic and standardized solution to the disease is warranted.

Several studies advocated the use of antiosteoporotic therapy, including calcium tablets, vitamin D, and calcitonin [11–14]. However, oral medication has the characteristics of low bioavailability and potential complications with longer times of usage. ZA is one of the most widely approved bisphosphonates due to its desired clinical effect on improving bone mass, relieving pain symptoms, and preventing additional fractures [15]. As the third generation of bisphosphonate drugs, ZA increases bone mass by binding to hydroxyapatite on the bone surface and blocking the mevalonate pathway to inhibit osteoclast-mediated bone resorption [16]. Moreover, a ZA infusion had particular advantages of improving bone metabolism indexes, the long-lasting effect of a once-yearly injection, and good compliance [17, 18], which provided insights into a comprehensive procedure for OVCFs. Recently, ZA combined with PKP/PVP treatment has been applied in patients with OVCFs in several preliminary studies, and the authors stated that it might be a valuable procedure in OVCF management. However, the long-term clinical outcome and standard usage plan remain unknown and rely more on personal experience [19]. Moreover, mild to severe complications are also reported occasionally, such as flu-like symptoms, postoperative leakage, osteonecrosis of the jaw, and atrial fibrillation. Indeed, an acute-phase response after ZA was observed in a number of papers [20]. Thus, systematic analyses of combination therapy are lacking. To date, several RCTs have compared PKP/PVP combined with ZA to the conservative PKP/PVP treatment using different assessments. We conducted this meta-analysis to provide better evidence of the efficacy and safety of PKP/PVP combined with ZA in treating OVCFs and proposed a protocol for its application in clinical practice.

## 2. Materials and Methods

The present meta-analysis was conducted based on the PRISMA 12 reporting guidelines for the meta-analysis of intervention trials [21].

### 2.1. Criteria for Including Studies

All RCTs of ZA combined with PVP/PKP compared to single PVP/PKP for the management of patients with OVCFs were included in this study. The clinical outcomes were pain symptoms assessed using visual analog scores (VAS) and the Oswestry Disability Index (ODI). Radiological outcomes consisted of the Cobb angle and vertebral body height (VBH). Bone metabolism indexes were evaluated using the bone mineral density (BMD), *β*-isomerized C-terminal telopeptide of type I collagen (*β*-CTX), N-terminal propeptide of type I collagen (PINP), and N-terminal molecular fragment (N-MID) levels.

### 2.2. Criteria for Excluding Studies

Studies of other treatments were excluded. Non-RCTs, clinical trials with fewer than 10 patients, cross-sectional studies, animal studies, case reports, comments, and reviews were excluded.

### 2.3. Database Searches

Electronic database searches were conducted from database inception to November 2020, including the Cochrane Library, PubMed, Web of Science, and Embase. The search terms on PubMed were as follows: “osteoporosis” (MeSH Terms), “fractures, bone” (MeSH Terms), and “zoledronic acid” (MeSH Terms). The search strategy was determined for each database. In addition, the language was restricted to English, with no limitation on subheadings. We searched reference lists of the identified papers to explore other studies, and trials not covered in the databases mentioned above were additionally searched once identified.

### 2.4. Data Collection and Analysis

The results were managed using Endnote X7 software, and duplicate studies were deleted by two well-trained authors with a sufficient understanding of this study. Next, two authors reviewed the abstracts and full texts of the included studies and selected the relevant information independently. Any disagreements were resolved by the third author. Data were independently extracted from selected studies by two authors who ultimately reached an agreement. Information for each eligible study included author information, publication year, country, methods of randomization and blinding, data sources, sample sizes, detailed interventions, treatment course, outcomes, follow-up duration, and adverse events. We contacted the relevant authors of the trials to obtain additional original data when necessary. The meta-analysis was performed using RevMan 5.3 software. Statistical heterogeneity was assessed using the *I*^2^ test and *P* value to quantify the inconsistencies within the included studies. A study had no heterogeneity with an *I*^2^ value less than 50%; otherwise, a value greater than 50% was regarded as significant heterogeneity. A fixed effects model was adopted if *I*^2^ ≤ 50%; otherwise, a random effects model was used. For continuous data, the mean difference (MD) was calculated. When the same outcomes were measured using different methods, we applied the standardized mean differences (SMDs) of 95% CIs in the meta-analysis. If significant heterogeneity was detected within studies, a subgroup analysis was performed by sequentially removing one study.

## 3. Results

### 3.1. Literature Search

**Figure 1:**
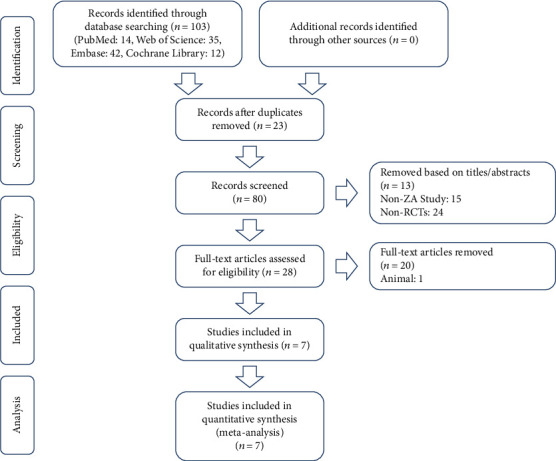
Flow diagram of the study selection process for the meta-analysis.

First, 103 studies were identified. Afterwards, we reviewed the abstracts and titles of the included studies, selected the relevant information, and removed duplicates independently, resulting in 96 studies. Finally, 7 RCTs were included after reading the full text (Figure 1). The characteristics of the included trials are shown in Table 1.

### 3.2. Risk of Bias

**Figure 2:**
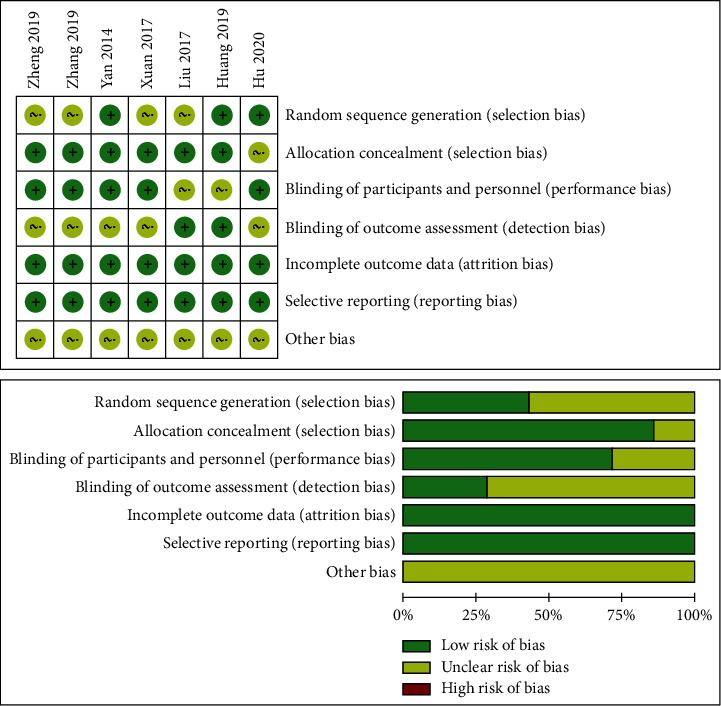
The methodological quality of the included studies. Risk of bias summary (a) and risk of bias graph (b): +: low risk of bias; −: high risk of bias; and ?: unclear risk of bias.

Of the 7 included studies, all studies were considered to have a low risk of bias. Random sequence generation was reported in 3 studies, allocation concealment in 6, blinding of participants and personnel in 5, and blinding of outcome assessment in 2. As shown in Figure 2, incomplete outcome data and selective reporting were not found in the 7 studies.

### 3.3. Clinical Parameters

#### 3.3.1. VAS Score

**Figure 3:**
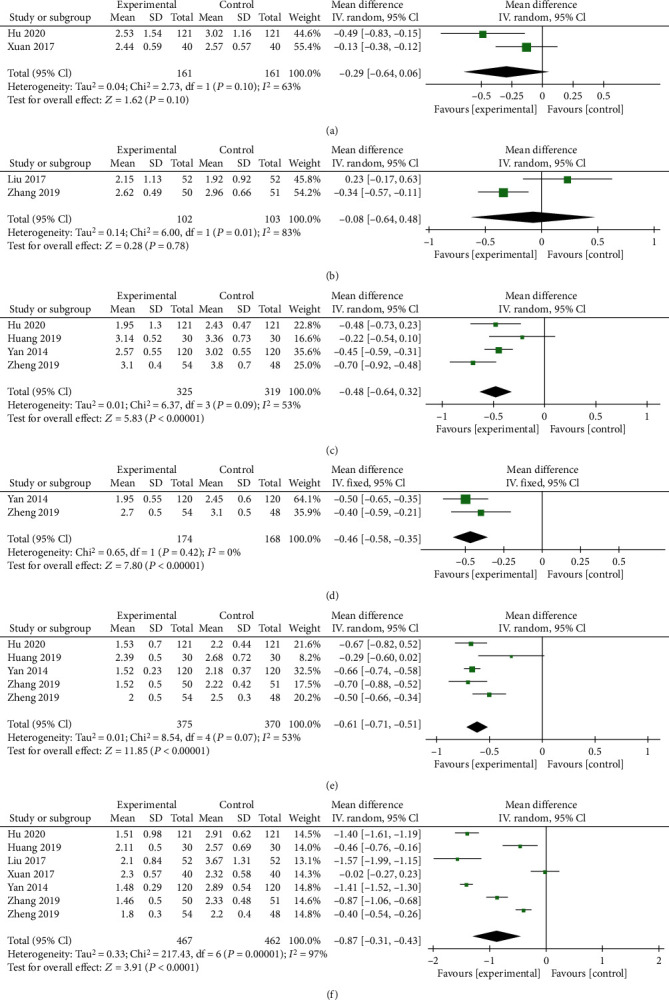
Forest plots of VAS scores recorded at 3 d (a), 1 w (b), 1 mo (c), 3 mo (d), 6 mo (e), and 12 mo (f) after the interventions. MD: mean difference; CI: confidence interval.

Seven trials including 929 subjects reported VAS scores. As shown in Figure 3, VAS scores were divided into 6 subgroups according to different follow-up time points. A random effects model was utilized when significant heterogeneity in subgroup differences was observed (*I*^2^ > 50%); otherwise, a fixed effects model was used. No statistically significant differences were found at the 3 d (*P* = 0.10, Figure 3(a)) and 1 w follow-ups (*P* = 0.78, Figure 3(b)). In contrast, significant differences were observed after interventions at follow-up times of 1 mo, 3 mo, 6 mo, and 12 mo (*P* < 0.00001, Figure 3(c); *P* < 0.00001, Figure 3(d); *P* < 0.00001, Figure 3(e); and *P* < 0.0001, Figure 3(f), respectively).

#### 3.3.2. ODI Score

**Figure 4:**
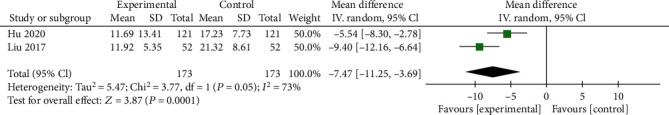
Forest plots of ODIs at the 12-month follow-up after the interventions. MD: mean difference; CI: confidence interval.

As shown in Figure 4, only 2 trials reported the ODI, including 346 subjects. A random effects model was utilized (*I*^2^ > 50%). A significant difference was observed at the 12 mo follow-up (*P* = 0.0001), with substantial heterogeneity (*I*^2^ = 73%, *P* = 0.05), which may be related to the limited number of studies analyzed.

### 3.4. Radiological Outcomes

**Figure 5:**
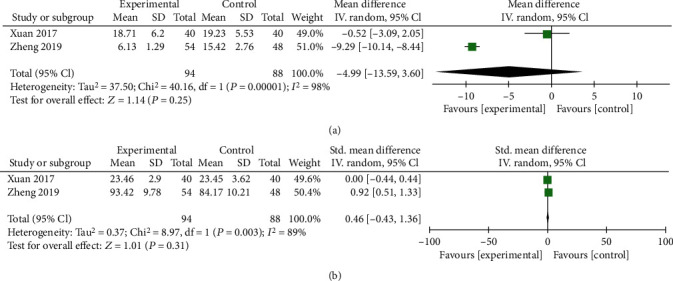
Forest plots of Cobb angles (a) and VBH (b) at the 12-month follow-up after the interventions. MD: mean difference; CI: confidence interval.

Two trials including 182 subjects reported the Cobb angle and VBH. As shown in Figure 5, no significant differences were observed in Cobb angle (*P* = 0.25, Figure 5(a)) and VBH (*P* = 0.31, Figure 5(b)) after interventions at the 12 mo follow-up. The random effects model was used due to the high heterogeneity. High heterogeneity may be attributed to the limited number of included trials. Nevertheless, ZA combined with PKP showed comparable efficacy with PKP alone in changing the Cobb angle and VBH.

### 3.5. Bone Metabolism Indexes

#### 3.5.1. BMD

**Figure 6:**
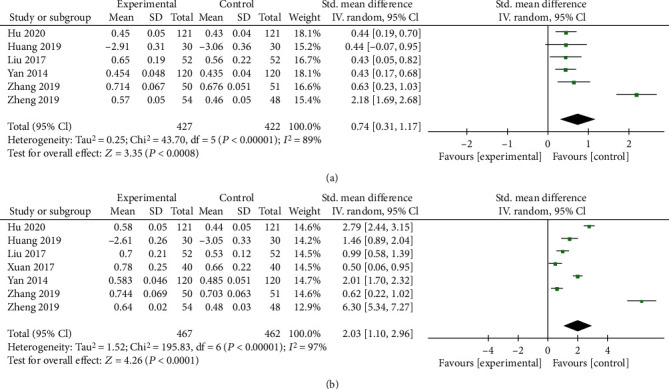
Forest plots of BMD at 6 mo (a) and 12 mo (b) after the interventions. MD: mean difference; CI: confidence interval.

All 7 trials including 929 subjects reported BMD. As shown in Figure 6, 2 subgroups were established due to the use of different follow-up time points. Significant differences were observed after the interventions at follow-up periods of 6 mo (*P* = 0.0008, Figure 6(a)) and 12 mo (*P* < 0.0001, Figure 6(b)), along with high heterogeneity. Thus, the random effects model was used. High heterogeneity may be due to the limited number of included trials, and more studies are needed in the future to analyze sources of heterogeneity. Nevertheless, ZA combined with PKP showed desirable long-term potential efficacy in improving BMD.

#### 3.5.2. *β*-CTX

**Figure 7:**
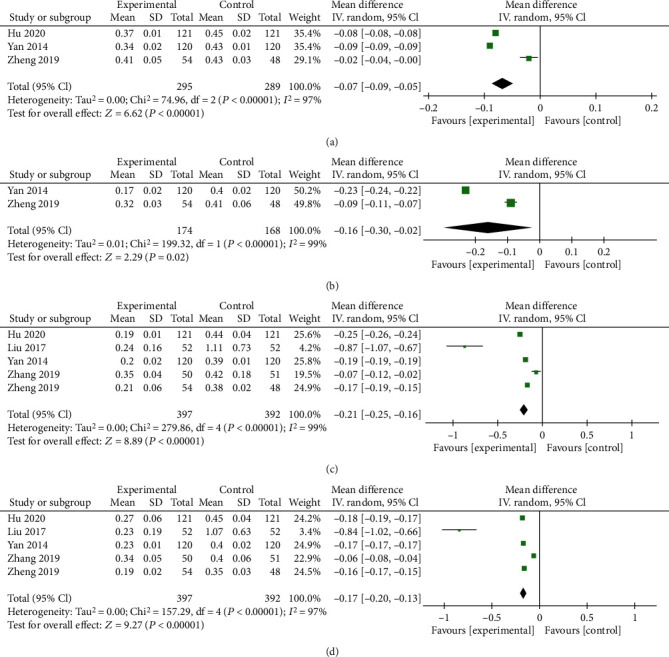
Forest plots of *β*-CTX levels at 1 mo (a), 3 mo (b), 6 mo (c), and 12 mo (d) after the interventions. MD: mean difference; CI: confidence interval.

Five trials including 789 subjects reported *β*-CTX levels, and 4 subgroups were generated due to the use of different follow-up time points (Figure 7). Significant differences were observed at follow-up periods of 1 mo, 3 mo, 6 mo, and 12 mo (*P* < 0.00001, Figure 7(a); *P* = 0.02, Figure 7(b); *P* < 0.00001, Figure 7(c); and *P* < 0.00001, Figure 7(d), respectively). The random effects model was used due to high heterogeneity. Then, a sensitivity analysis was conducted, and no trial had a decisive effect on the heterogeneity. According to the results, ZA combined with PKP showed reliable long-term efficacy in altering *β*-CTX levels.

#### 3.5.3. N-MID

**Figure 8:**
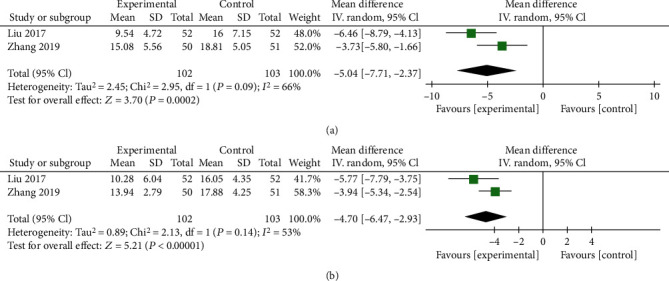
Forest plots of N-MID levels at 6 mo (a) and 12 mo (b) after the interventions. MD: mean difference; CI: confidence interval.

Two trials including 205 subjects reported N-MID levels, and 2 subgroups were established due to the use of different follow-up time points (Figure 8). Significant differences were observed at 6 mo (*P* = 0.0002, Figure 8(a)) and 12 mo (*P* < 0.00001, Figure 8(b)). The random effects model was used due to moderate heterogeneity. According to the results, ZA combined with PKP showed reliable long-term efficacy in altering the N-MID levels.

#### 3.5.4. PINP

**Figure 9:**
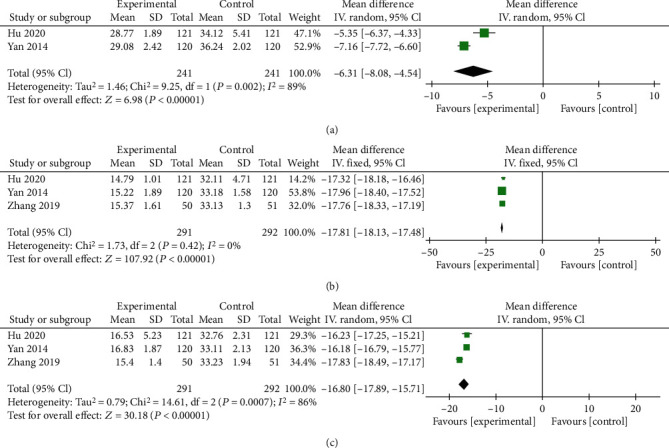
Forest plots of PINP levels at 1 mo (a), 6 mo (b), and 12 mo (c) after the interventions. MD: mean difference; CI: confidence interval.

Four trials including 583 subjects reported PINP levels, and 3 subgroups were generated due to the use of different follow-up time points (Figure 9). Significant differences were detected at the 1 mo, 6 mo, and 12 mo follow-ups (*P* < 0.00001, Figure 9(a); *P* < 0.00001, Figure 9(b); and *P* < 0.00001, Figure 9(c), respectively). According to the results, ZA combined with PINP showed reliable long-term efficacy in changing the N-MID levels.

### 3.6. Side Effects

**Figure 10:**
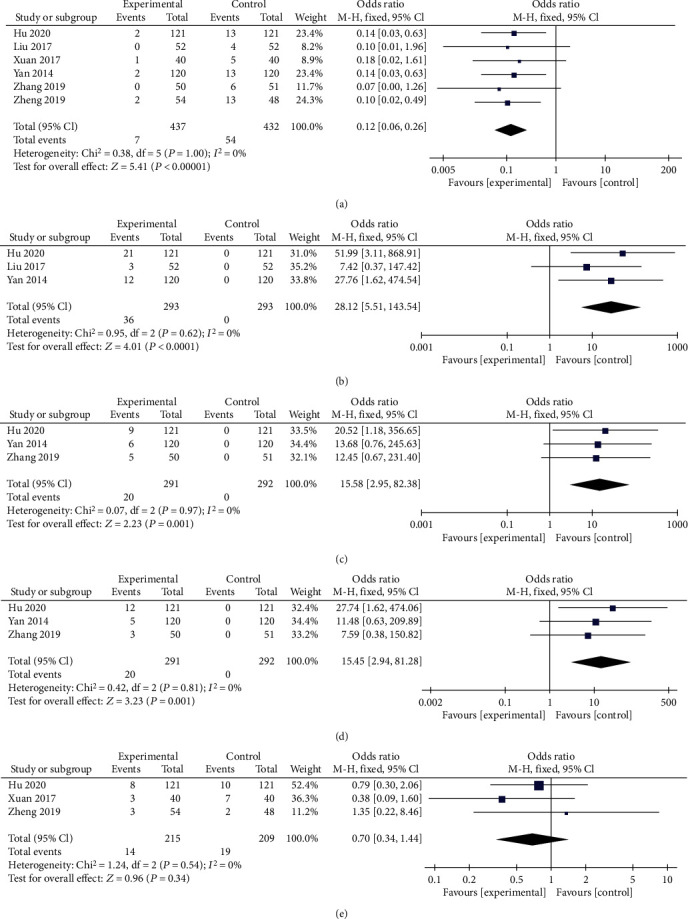
Forest plots of side effects after the interventions. Side effects included recurrent fractures (a), fever (b), flu-like symptoms (c), arthralgia or myalgia (d), and postoperative leakage (e). MD: mean difference; CI: confidence interval.

All trials reported side effects, and 5 subgroups were generated according to different types (Figure 10). Significant differences in recurrent fractures, fever, flu-like symptoms, and arthralgia or myalgia were observed (*P* < 0.00001, Figure 10(a); *P* < 0.0001, Figure 10(b); *P* = 0.001, Figure 10(c); and *P* = 0.001, Figure 10(d), respectively). However, no significant difference in postoperative leakage (*P* = 0.34, Figure 10(e)) was observed between the two groups. The fixed effects model was used due to no or low heterogeneity. As shown above, ZA combined with PKP produced more side effects, except for postoperative leakage.

### 3.7. Sensitivity Analysis

A sensitivity analysis was conducted to evaluate the effect of individual studies on the overall outcome by sequentially removing studies. A sensitivity analysis of VAS scores was conducted, and one trial by Zheng including 102 subjects had a decisive effect on the heterogeneity, without influencing the statistical results. After removing the study, the heterogeneity of the VAS score at 1 mo decreased to *I*^2^ = 0%, and the heterogeneity of the VAS score at 6 mo decreased to *I*^2^ = 45%, which indicated a stable result that was consistent with the subgroup analysis.

## 4. Discussion

The problem of osteoporosis tends to be more serious; thus, it has aroused wide concern throughout society. As the main complication of osteoporosis, conservative treatments for OVCFs have limited effects with many complications, especially for elderly individuals. In recent years, with the development of minimally invasive spinal surgery, the rapid and improved effect of PKP/PVP on the treatment of OVCFs has been accepted by a large number of scholars. Ideal management for OVCFs should satisfy the following conditions: long-term symptom improvement and lasting kyphotic deformity correction [1]. As minimally invasive procedures, PKP/PVP quickly supports the structure and restores the height of the vertebral body, corrects kyphosis, and relieves pain. During the process, spinal needles are placed into fractured vertebral bodies, and bone cement is injected inside. Intraoperative fluoroscopy and skillful techniques ensure smooth operations to avoid complications, such as bone cement leakage. Specifically, PKP is performed with a balloon, which is inflated to create an intravertebral body cavity and better correct kyphosis. Although similar outcomes have been reported [22, 23], few studies have claimed the possible advantages of PKP in terms of broader indications and restoring vertebral height [1]. Nevertheless, several complications must not be ignored, including postoperative pain and additional vertebral body fracture. The lack of long-term efficacy may be explained by the additional loading of injected bone cement and the natural progression of osteoporosis. Thus, integral treatment must be developed to optimize efficacy and avoid side effects. To the best of our knowledge, this study is the first meta-analysis to evaluate the efficacy and safety of ZA combined with PKP or PVP treatments for OVCFs.

**Figure 11:**
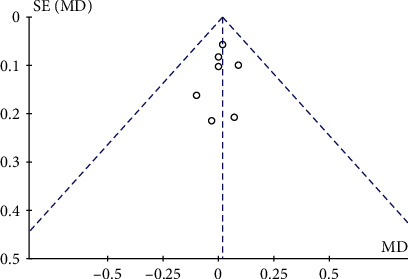
Funnel plot showing publication bias for studies comparing VAS scores between the two groups at the baseline. MD: mean difference; SE: standard error.

We conducted a search in electronic databases, including Cochrane Library, PubMed, Web of Science, and Embase, to collect all RCTs of ZA combined with PKP or PVP for the management of patients with OVCFs. Overall, the included RCTs were performed in China. The limited number of countries analyzed may cause bias; however, the techniques have been extensively utilized in China for twenty years since they were first proposed [8], and thus, surgeons have mastered the procedures. In addition, the sample size of each study was sufficient. The present meta-analysis was based on the PRISMA 12 reporting guidelines in accordance with the previous study [24] which maintained the reliability of the method. Therefore, with definite results from funnel plots comparing VAS scores between the two groups at baseline (Figure 11), the accuracy and reliability of the pooled results were rather convincing.

As a well-tolerated bisphosphonate, ZA has a high affinity for hydroxyapatite on the surface of bone, which specifically inhibits osteoclasts and bone absorption, retards bone loss, and improves bone mass [25]. Specifically, the biological process is related to inhibiting farnesyl pyrophosphate synthase, an enzyme in the mevalonate pathway, and subsequently preventing protein prenylation in osteoclasts to inhibit osteoclast-mediated bone resorption [16]. In addition, ZA is durable, has a rapid effect, and obviously improves bone density. Indeed, ZA has been approved and widely utilized for osteoporosis to relieve pain and prevent fractures of the vertebrae and other nonvertebral osseous structures [26, 27]. To date, combination treatment with ZA and PKP/PVP has been applied in patients with OVCFs and primary sequelae, and the authors stated that it might be a valuable procedure in OVCF management. However, the long-term effect of combination therapy remains unknown, and the acute-phase response after ZA should not be neglected. Moreover, a consensus on the dose and timing of ZA treatment has not been established. Thus, we aimed to provide better evidence of the efficacy and safety of PKP/PVP combined with ZA in treating OVCFs, as well as to propose a protocol for its application in clinical practice.

All RCTs included reported VAS scores, indicating the important role of analgesia in treatments. In the present study, no significant differences in VAS scores were observed between the two groups at the 3-day and 1-week follow-ups. However, significant differences in VAS scores were identified at the 1-month, 3-month, 6-month, and 12-month follow-ups after the operation. PKP/PVP exerted the desired effect on relieving pain within 1 week; moreover, the addition of ZA produced better long-term outcomes for pain intensity. The underlying mechanism by which PKP/PVP reduces pain remains unclear [28]. Nevertheless, a few compelling mechanisms have been proposed: heat caused by bone cement could cauterize nerve endings [29] and stabilize vertebral bodies [30, 31], resulting in peripheral nerve ischemia and necrosis by embolizing blood vessels [32]. Despite the doubts of a correlation between pain relief and radiological outcomes, several studies confirmed the efficacy in the early stage [33–35]. However, a certain number of patients still complained of mild residual pain, and surgical treatment is presumed to only relieve acute pain caused by the fracture but not the discomfort caused by osteoporosis. Additionally, fascia injury and injected bone cement were identified as risk factors for postoperative residual pain [36, 37]. Moreover, the progression of osteoporosis itself is a major cause of chronic pain. ZA was proven to alleviate pain symptoms in several trials [38, 39]. Thus, ZA combined with PKP/PVP exerted reliable analgesic effects, not only in the early stage but also in long-term observations.

ZA was originally used to treat osteoporosis, and researchers have focused on related bone metabolism indexes that could objectively and truly show efficacy. As a result, significant differences in BMD, *β*-CTX, PINP, and N-MID levels were observed between the two groups. Although these indicators remained steady or even improved after treatment of PKP/PVP alone, the incidence of recurrent fractures was significantly higher in the control group (*P* < 0.05). Improvements in the corresponding indicators were observed in the ZA group, along with a rather low fracture rate, indicating that this antiosteoporosis agent was essential for treatment efficacy. As a favorable indicator of bone resorption activity, a stable, low level of *β*-CTX was observed, inhibiting the activity of osteoclasts [40]. Moreover, P1NP and N-MID levels are sensitive markers of fractures and reliable in outcome assessments. ZA was proven to increase bone density and prevent fractures in numerous studies [41–43]. Hence, better bone metabolism indexes were undoubtedly obtained in the ZA with PKP/PVP group, which also explained the effect of long-term analgesia and inhibition of refracture. In addition, the trends for the indexes persisted at 12 months, indicating the stable and durable effect of the ZA injection. Hu et al. revealed that ZA treatment exerted a good inhibitory effect after 12 months [32].

Notably, 2 studies performed follow-up of the radiological results, and no significant differences were detected in the Cobb angle and VBH at 12 mo after the operation. PKP may have the advantage of restoring vertebral height due to the use of a balloon. However, scholars have questioned the difference between PKP and PVP; moreover, the use of the prone position during surgery may be an important reason for vertebral reduction [1, 44]. Overall, few studies have focused on the radiological results, and further research is needed. Nevertheless, controversy exists regarding whether improved vertebral body height correlates with clinical outcomes [22, 23], and the present study failed to address this issue.

Among the included studies, the dosage of ZA was uniform, and the duration was within 3 days in the perioperative period. The ZA group had significantly higher rates of fever, flu-like symptoms, arthralgia, and myalgia (*P* < 0.05), but not postoperative leakage (*P* > 0.05). However, the side effects were quite well tolerated, as symptoms disappeared after symptomatic treatment or naturally within 3 days. Severe complications, such as osteonecrosis of the jaw [45] and atrial fibrillation [46], were not observed. Additionally, ZA had no effect on postoperative leakage. Overall, the additional ZA injection was safe and reliable.

### 4.1. Limitations

To the best of our knowledge, this meta-analysis is the first to evaluate the effectiveness of ZA combined with PKP/PVP on OVCFs. However, the study had some limitations. First, the number of included studies was quite limited. Second, all studies were limited to English and were conducted in one country, which may lead to language bias. Third, studies comparing the combination of ZA with PKP/PVP with other antiosteoporotic therapies are lacking, and further studies are needed.

## 5. Conclusions

ZA combined with PVP/PKP not only exerted identical analgesic effects to PVP/PKP alone but also produced even better pain relief during long-term follow-up. In addition, the combination therapy significantly prevents complications with well-tolerated drug reactions and had advantages of long-term analgesic effects with improved bone metabolism indexes. Thus, ZA combined with PVP/PKP is a valuable, safe, and standard therapy for patients with OVCFs.

## Figures and Tables

**Table 1:** Characteristics of all the trials included in the meta-analysis.

Study	Country	Sample size		Age (years)		Interventions		ZA dose and timing	General treatments
		E	C	E	C	E	C		
Liu et al. (2017)	China	52	52	67.7 ± 7.6	70.9 ± 10.5	PKP+ZA	PKP	5 mg/100 ml, <15 min 2 days after the operation	Rifampicin tablets,120 mg, oral; dexamethasone, 5 mg, injection; before ZA
Zheng et al. (2019)	China	54	48	74.1 ± 6.4	73.2 ± 7.3	PKP+ZA	PKP+physiological saline	5 mg/100 ml 1 day after the operation	Calcium carbonate, 750 mg/d, oral; calcitriol, 0.4 *μ*g/d, oral; pre- and postoperation
Hu et al. (2020)	China	121	121	62.6 ± 7.2	67.5 ± 4.1	PVP+ZA	PVP	5 mg/100 ml 2 days prior to the operation	Calcium carbonate/vitamin D3 tablets, 600 mg/d, oral; pre- and postoperation
Zhang et al. (2019)	China	50	51	64.6 ± 6.7	64.0 ± 7.5	PKP+ZA	PKP	5 mg/100 ml, >15 min 2 days prior to the operation	Calcium carbonate, 3600 mg/d, oral; calcitriol, 0.25 *μ*g/d, oral; 1 year after the operation
Huang et al. (2019)	China	30	30	76.1 ± 8.3	74.4 ± 9.1	PKP+ZA	PKP	5 mg/100 ml 3 days after the operation	Calcium, 600 mg/2 d, oral; postoperation
Xuan et al. (2017)	China	40	40	70.5 ± 4.9	72.3 ± 4.6	PKP+ZA	PKP	5 mg/100 ml, 1 day after the operation	Calcium, 600 mg/d, oral; calcitriol, 0.25 *μ*g/d, oral; 1 year after the operation
Yan et al. (2014)	China	120	120	ND		PVP+ZA	PVP	5 mg/100 ml, 3 days after the operation	Calcium carbonate/vitamin D3 tablets, 600 mg/d, oral; 6 months after the operation

E: experimental group; C: control group; ND: the study did not report this information.

## Data Availability

The datasets used and/or analyzed during the present study are available from the corresponding author on reasonable request.

## References

[1] Ma X. L., Xing D., Ma J. X., Xu W. G., Wang J., Chen Y. (2012). Balloon kyphoplasty versus percutaneous vertebroplasty in treating osteoporotic vertebral compression fracture: grading the evidence through a systematic review and meta-analysis. *European Spine Journal*.

[2] Hiligsmann M., Dellaert B. G., Dirksen C. D. (2014). Patients' preferences for osteoporosis drug treatment: a discrete-choice experiment. *Arthritis Research & Therapy*.

[3] Shi G. H., Li P. C., Wei X. C. (2013). Progress on treatment of osteoporotic vertebral compression fracture. *Zhongguo Gu Shang*.

[4] Cauley J. A., Chalhoub D., Kassem A. M., Fuleihan G. (2014). Geographic and ethnic disparities in osteoporotic fractures. *Nature Reviews Endocrinology*.

[5] Klotzbuecher C. M., Ross P. D., Landsman P. B., Abbott T. R., Berger M. (2000). Patients with prior fractures have an increased risk of future fractures: a summary of the literature and statistical synthesis. *Journal of Bone and Mineral Research*.

[6] Takata S., Yasui N. (2001). Disuse osteoporosis. *The Journal of Medical Investigation*.

[7] Galibert P., Deramond H., Rosat P., Le Gars D. (1987). Preliminary note on the treatment of vertebral angioma by percutaneous acrylic vertebroplasty. *Neurochirurgie*.

[8] Garfin S. R., Yuan H. A., Reiley M. A. (2001). New technologies in spine: kyphoplasty and vertebroplasty for the treatment of painful osteoporotic compression fractures. *Spine (Phila Pa 1976)*.

[9] Zhong B. Y., He S. C., Zhu H. D. (2017). Risk prediction of new adjacent vertebral fractures after PVP for patients with vertebral compression fractures: development of a prediction model. *Cardiovascular and Interventional Radiology*.

[10] Shi C., Zhang M., Cheng A. Y., Huang Z. F. (2018). Percutaneous kyphoplasty combined with zoledronic acid infusion in the treatment of osteoporotic thoracolumbar fractures in the elderly. *Clinical Interventions in Aging*.

[11] Mansjur K. Q., Kuroda S., Izawa T. (2016). The effectiveness of human parathyroid hormone and low-intensity pulsed ultrasound on the fracture healing in osteoporotic bones. *Annals of Biomedical Engineering*.

[12] Semple D., Howlett M. (2016). A review of the use of calcium liquid versus calcium tablets for maintaining corrected calcium levels. *Archives Of Disease in Childhood*.

[13] Komm B. S., Chines A. A. (2012). An update on selective estrogen receptor modulators for the prevention and treatment of osteoporosis. *Maturitas*.

[14] Binkley N., Bone H., Gilligan J. P., Krause D. S. (2014). Efficacy and safety of oral recombinant calcitonin tablets in postmenopausal women with low bone mass and increased fracture risk: a randomized, placebo-controlled trial. *Osteoporosis International*.

[15] Burden A. M., Tadrous M., Calzavara A., Cadarette S. M. (2015). Uptake and characteristics of zoledronic acid and denosumab patients and physicians in Ontario, Canada: impact of drug formulary access. *Osteoporosis International*.

[16] Dunford J. E., Thompson K., Coxon F. P. (2001). Structure-activity relationships for inhibition of farnesyl diphosphate synthase in vitro and inhibition of bone resorption in vivo by nitrogen-containing bisphosphonates. *Journal of Pharmacology and Experimental Therapeutics*.

[17] Black D. M., Delmas P. D., Eastell R. (2007). Once-yearly zoledronic acid for treatment of postmenopausal osteoporosis. *The New England Journal of Medicine*.

[18] Lyles K. W., Colón-Emeric C. S., Magaziner J. S. (2007). Zoledronic acid and clinical fractures and mortality after hip fracture. *The New England Journal of Medicine*.

[19] Knopp-Sihota J. A., Newburn-Cook C. V., Homik J., Cummings G. G., Voaklander D. (2012). Calcitonin for treating acute and chronic pain of recent and remote osteoporotic vertebral compression fractures: a systematic review and meta-analysis. *Osteoporosis International*.

[20] Reid I. R., Gamble G. D., Mesenbrink P., Lakatos P., Black D. M. (2010). Characterization of and risk factors for the acute-phase response after zoledronic acid. *The Journal of Clinical Endocrinology and Metabolism*.

[21] Liberati A., Altman D. G., Tetzlaff J. (2009). The PRISMA statement for reporting systematic reviews and meta-analyses of studies that evaluate health care interventions: explanation and elaboration. *Journal of Clinical Epidemiology*.

[22] Röllinghoff M., Siewe J., Zarghooni K. (2009). Effectiveness, security and height restoration on fresh compression fractures--a comparative prospective study of vertebroplasty and kyphoplasty. *Minimally Invasive Neurosurgery*.

[23] Schofer M. D., Efe T., Timmesfeld N., Kortmann H. R., Quante M. (2009). Comparison of kyphoplasty and vertebroplasty in the treatment of fresh vertebral compression fractures. *Archives of Orthopaedic and Trauma Surgery*.

[24] Dong C., Sun Y., Qi Y., Zhu Y., Wei H., Di Wu C. L. (2020). Effect of platelet-rich plasma injection on mild or moderate carpal tunnel syndrome: an updated systematic review and meta-analysis of randomized controlled trials. *BioMed Research International*.

[25] Himelstein A. L., Foster J. C., Khatcheressian J. L. (2017). Effect of longer-interval vs standard dosing of zoledronic acid on skeletal events in patients with bone metastases: a randomized clinical trial. *JAMA*.

[26] Huang Z. F., Xiao S. X., Liu K., Xiong W. (2019). Effectiveness analysis of percutaneous kyphoplasty combined with zoledronic acid in treatment of primary osteoporotic vertebral compression fractures. *Pain Physician*.

[27] Liu Z., Li C. W., Mao Y. F. (2019). Study on zoledronic acid reducing acute bone loss and fracture rates in elderly postoperative patients with intertrochanteric fractures. *Orthopaedic Surgery*.

[28] Togawa D., Bauer T. W., Lieberman I. H., Takikawa S. (2003). Histologic evaluation of human vertebral bodies after vertebral augmentation with polymethyl methacrylate. *Spine (Phila Pa 1976)*.

[29] Heini P. F., Walchli B., Berlemann U. (2000). Percutaneous transpedicular vertebroplasty with PMMA: operative technique and early results. A prospective study for the treatment of osteoporotic compression fractures. *European Spine Journal*.

[30] Belkoff S. M., Mathis J. M., Jasper L. E., Deramond H. (2001). An ex vivo biomechanical evaluation of a hydroxyapatite cement for use with vertebroplasty. *Spine (Phila Pa 1976)*.

[31] Belkoff S. M., Mathis J. M., Jasper L. E., Deramond H. (2001). The biomechanics of vertebroplasty. The effect of cement volume on mechanical behavior. *Spine (Phila Pa 1976)*.

[32] Hu W., Wang H., Shi X. (2020). Effect of preoperative zoledronic acid administration on pain intensity after percutaneous vertebroplasty for osteoporotic vertebral compression fractures. *Pain Research & Management*.

[33] Ge J., Cheng X., Li P., Yang H., Zou J. (2019). The clinical effect of kyphoplasty using the extrapedicular approach in the treatment of thoracic osteoporotic vertebral compression fracture. *World Neurosurgery*.

[34] Wang H., Zhang Z., Liu Y., Jiang W. (2018). Percutaneous kyphoplasty for the treatment of very severe osteoporotic vertebral compression fractures with spinal canal compromise. *Journal of Orthopaedic Surgery and Research*.

[35] Clarençon F., Fahed R., Gabrieli J. (2016). Safety and clinical effectiveness of percutaneous vertebroplasty in the elderly (>/=80 years). *European Radiology*.

[36] Yang J.-S., Liu J.-J., Chu L. (2019). Causes of residual Back pain at early stage after percutaneous vertebroplasty: a retrospective analysis of 1,316 cases. *Pain Physician*.

[37] Yan Y., Xu R., Zou T. (2015). Is thoracolumbar fascia injury the cause of residual back pain after percutaneous vertebroplasty? A prospective cohort study. *Osteoporosis International*.

[38] Aitken D., Laslett L. L., Cai G. (2018). A protocol for a multicentre, randomised, double-blind, placebo-controlled trial to compare the effect of annual infusions of zoledronic acid to placebo on knee structural change and knee pain over 24 months in knee osteoarthritis patients - ZAP2. *BMC Musculoskeletal Disorders*.

[39] Cai G., Laslett L. L., Aitken D. (2018). Effect of zoledronic acid and denosumab in patients with low back pain and modic change: a proof-of-principle trial. *Journal of Bone and Mineral Research*.

[40] Xu Y., Wang Q., Hou G., Yao H., Zhao H. (2019). A dual-label time-resolved fluorescence immunoassay for screening of osteoporosis based on simultaneous detection of C-terminal telopeptide (*β*-CTX) and aminoterminal propeptide (P1NP) of type I procollagen. *Scandinavian Journal of Clinical and Laboratory Investigation*.

[41] Liang B. C., Shi Z. Y., Wang B. (2017). Intravenous zoledronic acid 5 mg on bone turnover markers and bone mineral density in East China subjects with newly diagnosed osteoporosis: a 24-month clinical study. *Orthopaedic Surgery*.

[42] Jeon O. C., Seo D. H., Kim H. S., Byun Y., Park J. W. (2016). Oral delivery of zoledronic acid by non-covalent conjugation with lysine-deoxycholic acid: in vitro characterization and in vivo anti-osteoporotic efficacy in ovariectomized rats. *European Journal of Pharmaceutical Sciences*.

[43] Bell K. J., Hayen A., Glasziou P. (2016). Potential usefulness of BMD and bone turnover monitoring of zoledronic acid therapy among women with osteoporosis: secondary analysis of randomized controlled trial data. *Journal of Bone and Mineral Research*.

[44] Hulme P. A., Krebs J., Ferguson S. J., Berlemann U. (2006). Vertebroplasty and kyphoplasty: a systematic review of 69 clinical studies. *Spine (Phila Pa 1976)*.

[45] Bagan J., Peydro A., Calvo J., Leopoldo M., Jimenez Y., Bagan L. (2016). Medication-related osteonecrosis of the jaw associated with bisphosphonates and denosumab in osteoporosis. *Oral Diseases*.

[46] Ilgezdi Z. D., Aktas I., Metin F. D. (2015). Acute effect of zoledronic acid infusion on atrial fibrillation development in patients with osteoporosis. *Anadolu Kardiyoloji Dergisi/The Anatolian Journal of Cardiology*.

